# Knockdown of long non-coding RNA CDKN2B-AS1 suppresses the progression of breast cancer by miR-122-5p/STK39 axis

**DOI:** 10.1080/21655979.2021.1962685

**Published:** 2021-08-10

**Authors:** Shaojie Qin, Mingliang Ning, Qingyuan Liu, Xiaoyun Ding, Yanbai Wang, Qilun Liu

**Affiliations:** a The Third Departments of Tumor Surgery, General Hospital of Ningxia Medical University, Yinchuan City, Ningxia, China; b Cerebrospinal Fluid Laboratory; General Hospital of Ningxia Medical University, Yinchuan City, Ningxia, China

**Keywords:** Lncrna cdkn2b-as1, miR-122-5p, stk39, human breast cancer

## Abstract

The lncRNAs have been made certain to take part in the development of most cancers in multiple ways. Here, our purpose is to making observation of the biological role and function of lncRNA CDKN2B-AS1 in human breast cancer. Twenty-eight pairs of breast cancer tissue and adjacent normal tissue from breast cancer patients were used to investigate the expression of CDKN2B-AS1 by qRT-PCR. And a lentivirus-shRNA guided CDKN2B-AS1 were to reduce its expression. The function of CDKN2B-AS1 was analyzed using a series of *in vitro* assays. Meanwhile, the xenograft model was used to further explicate the role of CDKN2B-AS1 in breast cancer. As for the results, there is a relative rich expression of CDKN2B-AS1 in breast cancer tissues compared with the corresponding adjacent normal tissues. Compared with the human breast epithelial cell line, the abundant expression of CDKN2B-AS1 in breast cancer cells were revealed as well. Then, knockdown CDKN2B-AS1 inhibited the malignant biological behaviors of MCF7 and T47D cells. In mechanism, CDKN2B-AS1 sponged the miR-122-5p to regulate STK39 expression. Furthermore, the inhibition effect with sh-CDKN2B-AS1 on breast cancer cells was alleviated by miR-122-5p inhibitor. Last, an i*n vivo* model also confirmed that knockdown CDKN2B-AS1 retarded the growth of breast cancer. Our data concluded that knockdown of CDKN2B-AS1 suppresses the progression of breast cancer by miR-122-5p/STK39 axis.

## 1. Introduction

Breast cancer (BC), as one of the highest degree of malignant cancers, seriously endangers the public health [1]. Only in 2020, among all newly diagnosed women cancer patients, the breast cancer alone accounts nearly for 30% [1–3]. The continuously increasing incidence of breast cancer caused a huge burden for public health [3,4]. Breast cancer is clinically classified into various subtypes, such as the luminal, human epidermal growth factor receptor 2 (HER2)-enriched, and triple-negative (TN) subtype [5]. A constant stream of breast cancer patients are putting the huge pressure on clinical treatment for breast cancer [4,6]. Nowadays, the therapeutic strategy for breast cancer is mainly surgery supplemented with chemotherapy [6,7]. Revealing the underlying mechanism about tumorigenesis and progression of breast is beneficial to clinical treatment. Thus, seeking a novel and effective therapeutic target in breast cancer is an urgent demand for breast cancer patients.

Recently, increasing researches have pointed that non-coding RNAs equally exert their biological function [8,9]. The long non-coding RNAs (lncRNAs) which participate in the progression in multiple ways have been proven in varieties of cancers [9,10]. Up to date, more and more studies have realized that lncRNAs could be an oncogene or a tumor suppressor in development of human tumors [9,11,12]. Under normal circumstances, lncRNAs perform as competing endogenous RNA (ceRNA) to adsorb miRNA as sponges [13,14]. The aberrant expression of lncRNAs would induce dysregulated miRNA expression, eventually impact on the cellular signaling pathways to affect cancer progression [14,15].

The lncRNA CDKN2B Antisense RNA 1 (CDKN2B-AS1) is located within the CDKN2B-CDKN2A (Cyclin Dependent Kinase Inhibitor 2B, Cyclin Dependent Kinase Inhibitor 2A) gene cluster at chromosome 9p21 [16]. It was reported that lncRNA CDKN2B-AS1 is associated with a number of pathologies, such as cardiovascular disease, Alzheimer’s disease and type-2 diabetes [17–19]. In breast cancer, several studies indicated that lncRNA CDKN2B-AS1 could be an independent bio-markers for diagnosis due to it has an aberrant expression pattern [20–22]. However, rare researches investigated how lncRNA CDKN2B-AS1 exerts its effects on breast cancer till date. The underlying mechanism about lncRNA functions in breast cancer remains enigmatic. Therefore, revealing the functional characteristic of lncRNA CDKN2B-AS1 in breast cancer is urgent and significant.

MicroRNAs (miRNAs, ~20 nt) belong to a class of non-coding RNAs that are highly conserved in evolution [23]. With the development of the tumors, the expression profile of miRNAs alteration occurs [23,24]. It has been verified that this alteration could promote and repress the progression of most cancers [25–27]. In general, miRNAs binds to the matched target 3ʹ-UTR of mRNA to restrict the target gene expression through forming a RNA-induced silencing complex (RISC) [28]. Besides, some studies also indicated that the combined mRNA would be spliced to degrade [28,29]. Either way, miRNAs regulate the expressions of genes on decreasing the targets protein level [28]. MiR-122-5p as an RNA gene is related to various diseases, including majorities of cancers like hepatocellular carcinoma (HCC), non-small cell lung cancer (NSCLC) and so on [30–32]. Also in breast cancer, miR-122-5p was involved in the progression or chemo-sensitizes of breast cancer [33].

Nevertheless, the miRNAs function often rely on the target downward protein [28]. Serine/Threonine Kinase 39 (STK39) encodes a serine/threonine kinase affecting cellular stress response pathway [34]. This kinase is closest implicated in oncogenesis, such as HCC, osteosarcoma and so on [34,35]. Previous researches have revealed that STK39 excited the mitogen-activated protein kinase (MAPK) pathway in tumorigenesis [36]. As is well known, MAPK signaling usually play a vital factor to participate in varieties of cancers [37]. Especially in breast cancer, MAPK signaling pathway regulates the cancer progression tightly [38]. Meanwhile, many studies reported that STK39 accelerated the development of breast cancer [39]. Hence, STK39 could regulate the development of breast cancer and reducing STK39 expression receded the malignancy of breast cancer.

To sum up, the following hypothesis is proposed that lncRNA CDKN2B-AS1 took part in regulating the progression of breast cancer. Herein, our aim and goal are to reveal the role and the mechanism of lncRNA CDKN2B-AS1 in human breast cancer. Through a systematical investigation of lncRNA CDKN2B-AS1, it would provide for clinical treatment of breast cancer with a potential therapeutic target and a novel diagnostic bio-marker.

## 2. Materials and methods

### 2.1 Tissue samples

The tumor tissue samples and matched adjacent normal tissues were obtained from breast cancer patients (28 females; age: 45–65 years’ old) at General Hospital of Ningxia Medical University between 2019 and 2020 with written informed consent. The clinical characteristics of patients with breast cancer are shown in Table 1. This study was reviewed and approved by the Ethics Review Committee of General Hospital of Ningxia Medical University and was performed in accordance with Declaration of Helsinki.

**Table 1.** The relationships between CDKN2B-AS1 expression and clinicopathological characteristics of breast cancer patients

Characteristics	Expression of		P value
	Low(n = 14) High(n = 14)		
Age			0.4302
≤ 50	4	6	
> 50	10	8	
Lymph node mestasis			0.7047
Yes	6	7	
No	8	7	
Pathological Staging			0.0530
I + II	8	3	
III + IV	6	11	
Distant metastasis			0.6857
M0	5	4	
M1	9	10	
ER			0.4450
Negative	5	7	
Positive	9	7	
PR			0.7047
Negative	7	6	
Positive	7	8	
HER2			0.7047
Negative	7	8	
Positive	7	6	

ER, estrogen receptor; PR, progesterone receptor. Low/high by the sample mean. Pearson χ2 test. *P < 0.05 was considered statistically significant.

### 2.2 Cells culture

Human normal breast basal epithelial cell, MCF10A, and human breast cancer cell lines, MCF7, T47D and MDA-MB-231 were purchased from Cell Bank of Type Culture Collection of Chinese Academy of Sciences (Shanghai, China). Medium selection for these cells was base DMEM medium with 10% FBS, 100 IU/mL penicillin, and 100 μg /mL streptomycin (Gibco, the US). All cells were incubated at 37°C, 5% CO_2_.

### 2.3 Cell transfections

MCF7 and T47D cells were infected with the lentivirus (sh CDKN2B-AS1 and sh NC, MOI = 25) to stably knockdown CDKN2B-AS1 in MCF7 and T47D cells. The lentivirus and infection regent HitransG A were obtained from Genechem (Shanghai, China). Through incubation with 2 mg/ml puromycin for 48 h, sh CDKN2B-AS1 and sh NC breast cancer cells were harvested by trypsin digestion and centrifugation. When cells reach at 50% confluence, cells were transfected with a miR-122-5p mimic (100 pmol), a miR-122-5p inhibitor (100 pmol) and their corresponding negative control were carried out as required in assigned experiment by using Lipofectamine™3000 (ThermoFisher, USA) to conduct follow-up experiments. All the miRNA mimic and inhibitor were synthesized from Invitrogen (Invitrogen, Shanghai, China).

### 2.4 Cell viabilities

The cell viabilities were assessed by the CCK-8 (Beyotime Institute of Biotechnology, Shanghai, China) solution and absorbance was measured at 450 nm through a Microplate Reader (Bio-Rad, USA). Briefly, planted 4000 transfected MCF7 cells or T47D cells in 96-well plates per well on day 0. After 6 hours and on Day1, Day2 and Day3, respectively, the absorbance was measured to determine the cell viability by adding 10 μl CCK-8 solution for another 4 hours at 37°C and 5% CO_2_.

### 2.5 Colony formation assays

About 600 cells were evenly planted into a 6-well plate for 10 days, then washed with PBS once, fixed with formaldehyde solution and stained with 0.1% crystal violet solution in turn. More than 50 cells were considered to a colony. The numbers of colonies were counted under an inverted microscope (Leica Microsystems, German).

### 2.6 Apoptotic cells

The apoptotic cells rates were analyzed by flow cytometry (FACScan, Beckman Coulter, USA) with an Annexin V-FITC/PI (Invitrogen, USA) assay. Briefly, all suspended and adherent cells were collected, and then resuspended and stained with 5 μl of Annexin V-FITC and 10 μl of PI for 15 min in dark. Finally, the apoptotic rate of different treatment groups was analyzed by flow cytometry.

### 2.7 Cell cycle

Cells were washed with PBS twice and fixed with 70% ethanol at 4°C overnight. After centrifugation, cells were washed and resuspended in 500 μL BD Pharmingen PI/RNase staining buffer (BD Biosciences). Then cells were incubated for 15 minutes at room temperature and analyzed on the same flow cytometer.

## 2.8 qRT-PCR

Total RNA was extracted with TRIzol reagent (Invitrogen). cDNAs were produced with the RNA templates by using a reverse transcription kit (Invitrogen). qRT-PCR analysis was performed on a CFX96 Thermal Cycler Dice^TM^ real-time PCR system with SYBR Premix Ex Taq II (TaKaRa, Dalian, China). GAPDH and U6 were used as reference gene, respectively. The primers and shRNA in this article are in Table 2. The relative expressions were calculated by using 2^−ΔΔCt^ method.

**Table 2.** The primers and shRNA in this article are as follow

Gene name	Primer sequence (5ʹ to3ʹ)
CDKN2B-AS1	Forward: 5ʹ-CTATCCGCCAATCAGGAGGC-3ʹ Reverse: 5ʹ-AAAAGGGACACTAGTCCGGC-3’
miR-122-5p	Forward: 5ʹ-TATTCGCACTGGATACGACACAAAC-3ʹ Reverse: 5ʹ-GCCCGTGGAGTGTGACAATGGT-3’
STK39	Forward: 5ʹ-CTTCTTGTGCCGTGAACCTCGT-3ʹ Reverse: 5ʹ-GCTCCTGAGATACACCATCTGC-3’
GAPDH	Forward: 5ʹ-ATCCACGGGAGAGCGACAT-3ʹ Reverse: 5ʹ-CAGCTGCTTGTAAAGTGGAC-3’
U6	Forward: 5ʹ-ACAGATCTGTCGGTGTGGCAC-3ʹ Reverse: 5ʹ-GGCCCCGGATTATCCGACATTC-3’
sh-CDKN2BAS1	5ʹ-GCAGTTGCTACAAGTTAGACTCGAGTTGCTACAAGTTAGTACGCTTTTT-3’
sh-NC	5ʹ-GGAGATATTCTTTCAAACCCTCATTCTTTCAAACCCTCCGCTTTTTT-3’

### 2.9 Luciferase reporter assays

All plasmids were made from Genepharma, China. We first insert the containing underlying binding site sequence of CDKN2B-AS1 Wild Type (WT) or CDKN2B-AS1 mutant type (MUT) and into a luciferase report gene vectors (pRL-TK, Promega) to establish the two types plasmid. Subsequently, miR-122-5p mimic or miR NC was co-transfected into MCF7 and T47D cells with reporter plasmids for 48 h. The relative luciferase activity was detected by Dual-Luciferase Reporter Assay System (Promega). About 50 ng renilla plasmid was used as an internal reference. The same approach applies to the STK39 Wild Type (WT) or STK39 mutant type (MUT) plasmid.

### 2.10 Western blotting

The protein is extracted by cell lysis (RIPA Lysis Buffer, Beyotime, Shanghai, China)) and denatured with loading buffer to load into 10% SDS-PAGE gels. Subsequently, the samples were separated and transferred to PVDF membrane (Bio-Rad, USA). Then, the primary antibody, anti-STK39 (1:1000, orb100341, biorbyt) and anti-GAPDH (1:1000, ab8245, abcam), was incubated after blocking with blocking buffer overnight. On the second day, HRP conjugated secondary antibodies were incubated after washing. Finally, the proteins were examined through chemiluminescence. GAPDH was a reference.

### 2.11 Xenograft mouse model

Ten female mice (8 weeks old) were divided into two groups (sh NC and sh CDKN2B-AS1) and amount of 5 × 10^6^ sh CDKN2B-AS1 and sh NC MCF7 cells were inoculated in the axils of each mouse. Tumor volume was measured every 3 days according to the following formula: volume = 1/2 × length × width^2^. Animal protocols, housing, and care were performed with the approval of the Ethics Committee of General Hospital of Ningxia Medical University and conducted according to the guidelines set forth in the National Institutes of Health’s (NIH) ‘Guide for the Care and Use of Laboratory Animals’ (8th edition).

### 2.12 Immunohistochemistry (IHC)

The dissected tumors were paraffin-embedded and cut into 4-μm sections. Through an antigenic repair process, the sections were incubated with antibodies, anti-Ki67 antibody (1:200, ab15580, abcam) or anti-STK39 (1:200, orb100341, biorbyt), at 4°C overnight. Subsequently, the sections were washed the primary antibodies away to incubate biotinylated secondary antibodies for 2 h at room temperature. IHC images were taken using an Olympus microscope after visualizing with diaminobenzidine substrate (Sigma-Aldrich, St Louis, MO, USA).

### 2.13 Statistical analysis

All statistical analyses were conducted using the SPSS statistical package (16.0, SPSS Inc. Chicago, IL). Unpaired student’s t test was used to compare the means of two groups of data. The data were shown as mean ± SD. P-values were calculated by ANOVA, with *P* < 0.05 considered as significant.

## 3. Results

In our study, we proposed that lncRNA CDKN2B-AS1 took part in regulating the progression of breast cancer. Thus, our aim and goal was to reveal the role and the mechanism of lncRNA CDKN2B-AS1 in human breast cancer. In this study, we first found that the different expression pattern of lncRNA CDKN2B-AS1 and miR-122-5p in breast cancers by detecting the collected clinical cancer samples. Subsequently, knockdown the inherently highly expressed lncRNA CDKN2B-AS1 with sh-RNA in order to investigate the function of CDKN2B-AS1. Through a series of functional and mechanism experiments, we concluded that lncRNA CDKN2B-AS1 acts as a miR-122-5p sponge to regulate the STK39 expression, and promotes breast cancer progression.

### 3.1 The aberrant expressions of lncRNA CDKN2B-AS1 in breast cancer tissues and cells.

**Figure 1.**
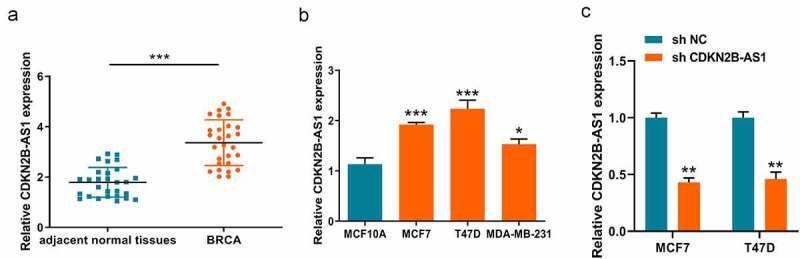
**The aberrant expressions of lncRNA CDKN2B-AS1 in breast cancer tissues and cells**. (a). The level of CDKN2B-AS1 in breast cancer tissues and adjacent normal tissues was examined by RT-qPCR; (b). The expression levels of CDKN2B-AS1 were detected by RT-qPCR in MCF10A and breast cancer cells; (c). The expression of CDKN2B-AS1 were detected by qRT-PCR after knockdown of CDKN2B-AS1 with shRNA. Data are presented as mean ± SD. n = 6, *P < 0.05, **P < 0.01, ***P < 0.001

Finding out the expression levels of CDKN2B-AS1 gave a hint on the role in development of breast cancer. Therefore, we initially detected the CDKN2B-AS1 expression in the 28 collected breast cancer tissues from breast patients. As shown in Figure 1a, CDKN2B-AS1 present a higher expression level in breast cancer tissues compared with the adjacent normal tissues. Due to CDKN2B-AS1 is upregulated in breast cancer tissues, we then detected the expression in breast cancer cells. Similarly, the level of CDKN2B-AS1 in breast cancer cells is consistent with the previous result in breast tissues. All the three breast cancer cells had the relative rich expression in comparison to the human normal breast basal epithelial cell (MCF10A). In Figure 1b, the expressions of CDKN2B-AS1 in MCF7 and T47D have more significant difference with the expression of MCF10A (***P < 0.001) compared with the difference between MDA-MB-231 and MCF10A (*P < 0.05). Therefore, we chose this two breast cancer cells with the shRNA system to conduct follow-up experiments. Knockdown of CDKN2B-AS1 with an shRNA lowered the CDKN2B-AS1 level in MCF7 and T47D cells (Figure 1c). All these results indicated the dysregulated expressions of CDKN2B-AS1 might participate in progression of breast cancer.

### 3.2 Knockdown of CDKN2B-AS1 repressed the MCF7 and T47D cell proliferation and induced the apoptosis.

**Figure 2.**
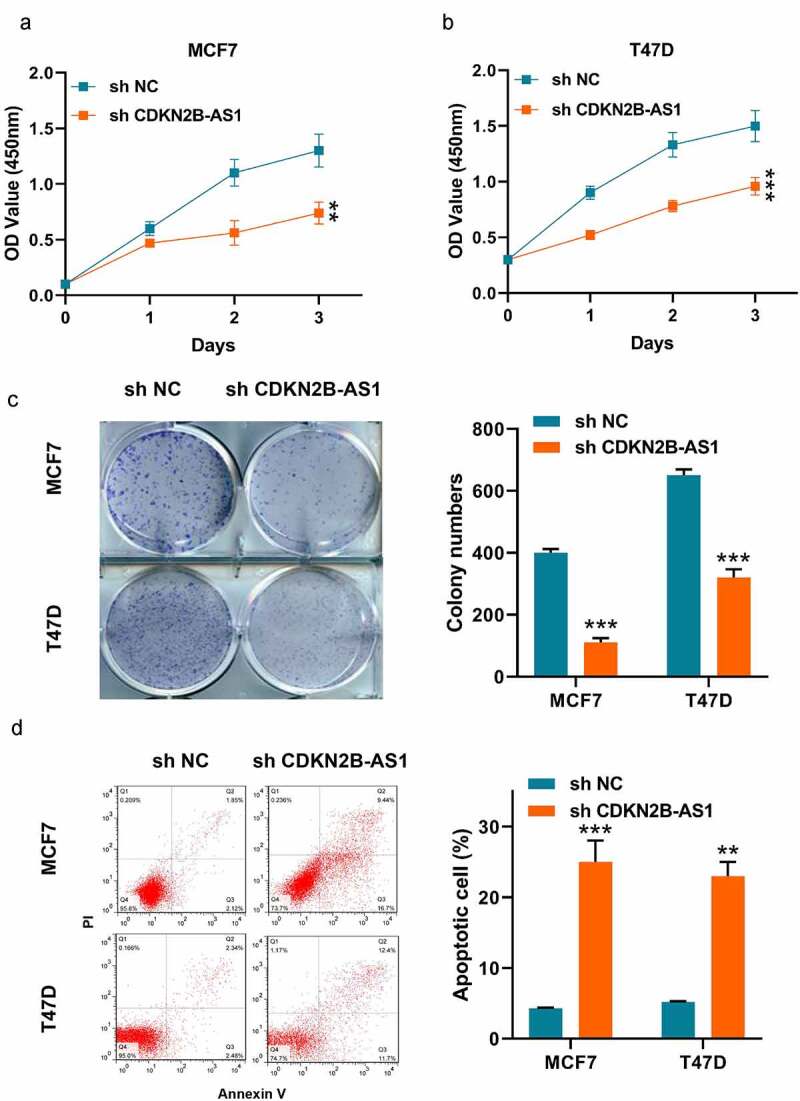
**Knockdown of CDKN2B-AS1 inhibited the breast cancer cell proliferation and induced apoptosis**. (a) and (b). The relative cell viability was detected by CCK-8 assay in MCF7 and T47D cells; (c). The cell proliferation was evaluated with the colony formation assay in MCF7 and T47D cells; (d). The cell apoptosis was evaluated by flow cytometry in MCF7 and T47D cells; Data are presented as mean ± SD. n = 6, **P < 0.01, ***P < 0.001

Then, we examined the effect of sh-CDKN2B-AS1 on the proliferation of the two cells. As shown in Figure 2a and 2b, the CCK-8 results showed that inhibition of CDKN2B-AS1 decreased the cell viability of MCF7 and T47D cells. Meanwhile, the clonality of the two cells were repressed by knockdown of CDKN2B-AS1 (Figure 2c). In addition, we detected the apoptosis of the two cancer cells as well. It was illustrated that lowering the expression of CDKN2B-AS1 would lead to induce the apoptosis (Figure 2d). From the above results, we could summarize that knockdown of CDKN2B-AS1 affected the MCF7 and T47D cellular functions.

### 3.3 MiR-122-5p is a target of lncRNA CDKN2B-AS1.

**Figure 3.**
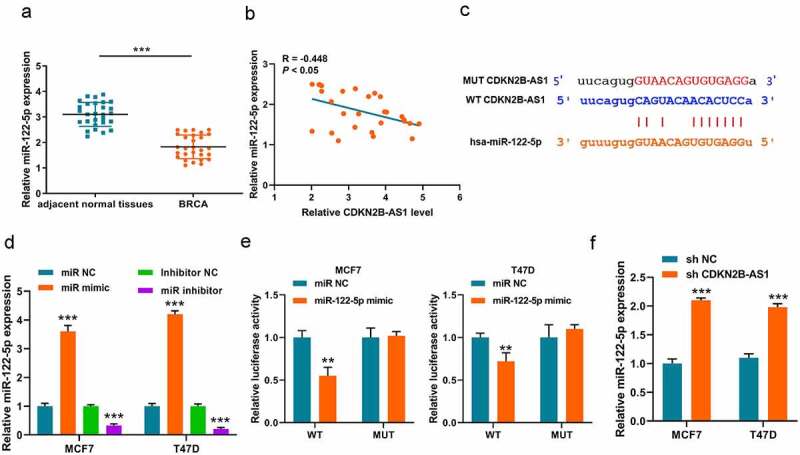
**MiR-122-5p is a target of CDKN2B-AS1**. (a). The expression levels of miR-122-5p in breast cancer tissues and adjacent normal tissues were detected by RT-qPCR; (b). The correlation between CDKN2B-AS1 and miR-122-5p was analyzed in breast cancer tissues (R = −0.448, *P* < 0.05); (c). The binding site between CDKN2B-AS1 and miR-122-5p; (B). Luciferase activity was examined in MCF7 and T47D cells; (d). The levels of miR-122-5p were detected by qRT-PCR in MCF7 and T47D cells; (e). Luciferase activity was examined in MCF7 and T47D cells; (f). The levels of miR-122-5p in MCF7 and T47D cells. Data are presented as mean ± SD. n = 5, **P < 0.01, ***P < 0,001

Upon most occasions, lncRNAs perform as ceRNA to adsorb miRNA as sponges. Thus, we first analyzed the underlying downstream miRNAs of lncRNA CDKN2B-AS1 on Online database (http://starbase.sysu.edu.cn/). Among the numerous of results, miR-122-5p is a potential target of CDKN2B-AS1. Thus, we also detected the expression of miR-122-5p in breast cancer tissues and a downward trend was discovered in breast cancer (Figure 3a). Through analyzing correlation between the expression pattern of lncRNA CDKN2B-AS1 and miR-122-5p in breast cancer, we found that there was a negative correlation (R = – 0.448, *P* < 0.05) in Figure 3b. Subsequently, to verified this interaction, we construct the luciferase reporter plasmid system (Figure 3c) and the miR-122-5p mimic, miR-122-5p inhibitor and respective negative control were transfected into MCF7 and T47D cells for elevating or repressing the miR-122-5p level (Figure 3d). The luciferase system results indicated that miR-122-5p could lower the luciferase activity of WT plasmid in MCF7 and T47D cells, but made no difference on MUT plasmid (Figure 3e). Finally, we verified that the expressions of miR-122-5p were elevated in sh-CDKN2B-AS1 MCF7 and T47D cells (figure 3f). All these results indicated that knockdown of CDKN2B-AS1 could elevate the expression of miR-122-5p.

### 3.4 STK39 is a target of miR-122-5p.

**Figure 4.**
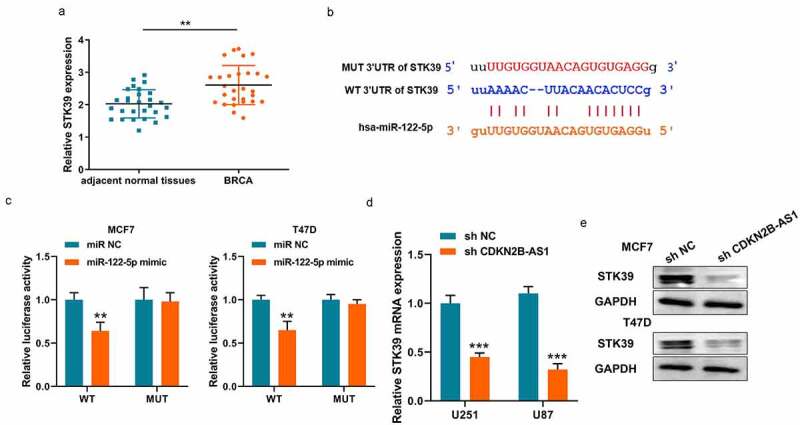
**STK39 is a target of miR-122-5p**. (a). The expression levels of STK39 in breast cancer tissues and adjacent normal tissues were detected by RT-qPCR; (b). The binding site between miR-122-5p and STK39; (c). Luciferase activity was examined in MCF7 and T47D cells; (B). Luciferase activity was examined in MCF7 and T47D cells; (d). The mRNA levels of STK39 in MCF7 and T47D cells; (e). The protein levels of STK39 in MCF7 and T47D cells. Data are presented as mean ± SD. n = 5, **P < 0.01, ***P < 0,001

Similarly, we also analyzed the underlying downstream targets of miR-122-5p on Online database. STK39 is a potential target of miR-122-5p and an upward trend was discovered in breast cancer (Figure 4a). The binding site and corresponding luciferase reporter plasmid were also verified the interaction between miR-122-5p and STK39 (Figure 4b). And it also illustrated that miR-122-5p only affected WT fluorescence intensity and doesn’t affect MUT intensity (Figure 4c). Meanwhile, the mRNA and protein level of STK39 were both downregulated when CDKN2B-AS1 expression was restricted in MCF7 and T47D cells (Figure 4d and 4e). Thus, CDKN2B-AS1 regulate the expressions of miR-122-5p and STK39 as a ceRNA sponge.

### 3.5 LncRNA CDKN2B-AS1 affected the biological behaviors of MCF7 and T47D cells through miR-122-5p/STK39 axis

**Figure 5.**
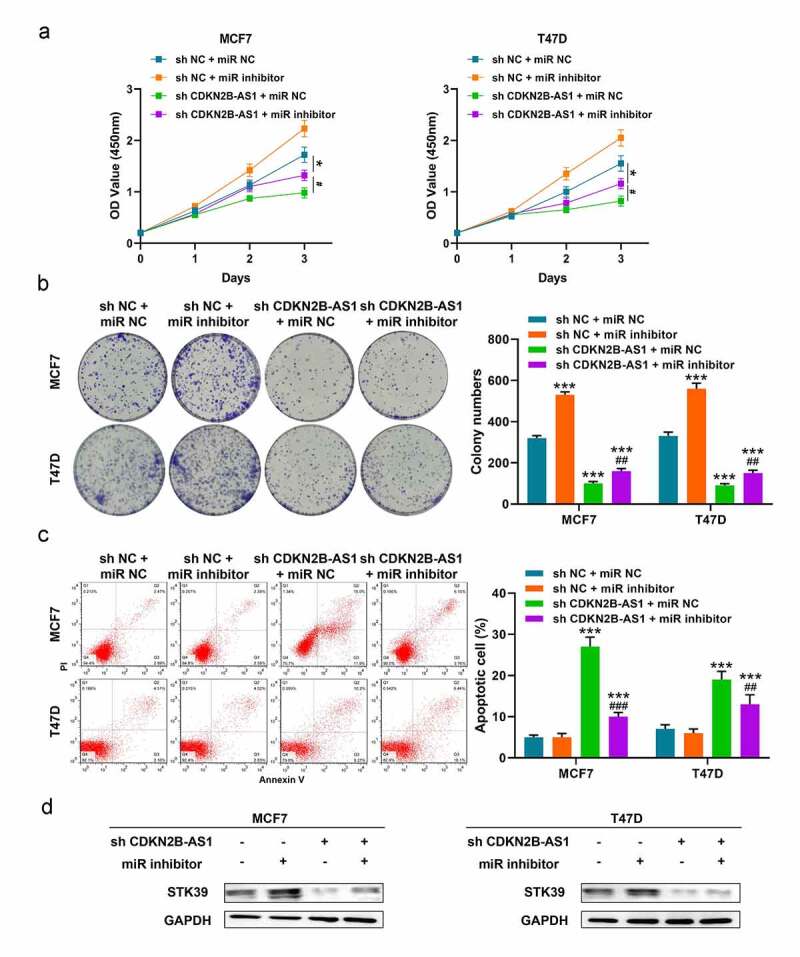
**LncRNA CDKN2B-AS1 affected the functions of MCF7 and T47D cells through miR-122-5p/STK39 axis**. (a). The relative cell viability was detected by CCK-8 assay; (b). The cell proliferation was evaluated with the colony formation assay; (c). The cell apoptosis was evaluated by flow cytometry; (d). The protein levels of STK39 in MCF7 and T47D cells. Data are presented as mean ± SD. n = 5, * indicated the difference compared with the group of sh NC + miR NC; # indicated the difference compared with the group of sh CDKN2B-AS1 + miR NC; *P < 0.05, ***P < 0.001, #*P* < 0.05, ##*P* < 0.01, ###*P* < 0.001

Based on previous results, we speculated that lncRNA CDKN2B-AS1 functions via miR-122-5p and STK39 and restricting the expression of miR-122-5p with miR inhibitor could alleviate the impact of sh-CDKN2B-AS1 in MCF7 and T47D cells. In Figure 5a and 5b, the inhibition of sh-CDKN2B-AS1 on cell viability and colony formation were relieved with miR-122-5p inhibitor in MCF7 and T47D cells. Likewise, miR-122-5p inhibitor also weakened the apoptosis occurrence when CDKN2B-AS1 were knockdown in MCF7 and T47D cells (Figure 5c). In addition, the protein level of STK39 was elevated after transfecting with miR-122-5p inhibitor in sh-CDKN2B-AS1 MCF7 and T47D cells (Figure 5d). Thus, we considered that CDKN2B-AS1 regulated tumor progression of MCF7 and T47D cells through miR-122-5p/STK39 axis.

### 3.6 The biological role of CDKN2B-AS1 was further clarified in the xenograft breast cancer model.

**Figure 6.**
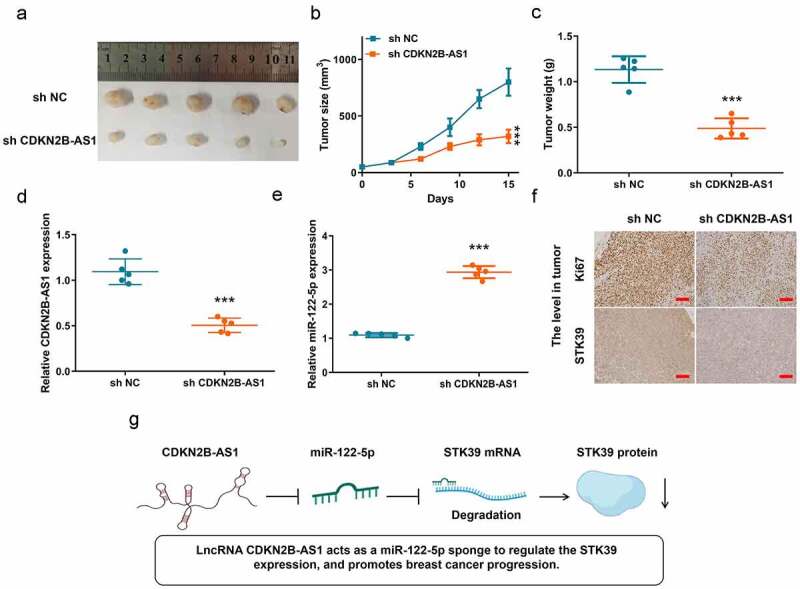
***In vivo* experiment confirmed that knockdown of CDKN2B-AS1 inhibited the progression of breast cancer**. (a). The image of tumors; (b). The tumor volume; (c). The tumor weight; (d) and (e). The levels of CDKN2B-AS1 and miR-122-5p in tumors; (f). The Ki67 level in tumor and the STK39 level in tumor, Scale bar: 50 µm; (g). The schematic diagram of mechanism. n = 5, ***P < 0.001

To further clarify the biological role of lncRNA CDKN2B-AS1 in breast cancer, we established a xenograft tumor model on BALB/C nude mice with sh-CDKN2B-AS1 MCF7 and sh-NC MCF7 cells. The tumors were collected form the mice on day 15 after inoculation (Figure 6a). In Figure 6b and 6c, inhibition of lncRNA CDKN2B-AS1 significantly suppressed the tumor growth and decreased tumor weight. Meanwhile, in sh-CDKN2B-AS1 tumors, the expressions of CDKN2B-AS1 and miR-122-5p were repressed and increased, respectively. For detecting the level of Ki67 and STK39, sh-CDKN2B-AS1 lowered both the expression in tumor (figure 6f). Hence, knockdown of CDKN2B-AS1 could retard the progression of breast cancer. To sum up, we found that CDKN2B-AS1 acts as a miR-122-5p sponge to regulate the STK39 expression, and promotes breast cancer progression (Figure 6g).

## 4. Discussions

In our study, we first revealed that CDKN2B-AS1 acted as a miR-122-5p sponge to regulate the STK39 expression, and promoted breast cancer progression. Our data confirmed that reducing the lncRNA CDKN2B-AS1 expression restrained the proliferation of MCF7 and T47D cells, and increased apoptotic rate of the two breast cancer cells. Moreover, we further uncovered that miR-122-5p mediated the effect of sh-CDKN2B-AS1 through altering STK39 expression. In addition, a xenograft model with MCF7 cells confirmed knockdown of CDKN2B-AS1 retarded the breast cancer progression. To sum up, these results support the conclusion that CDKN2B-AS1 participates in the development of breast cancer cells.

Based on our data, lncRNA CDKN2B-AS1 played an oncogenic factor in breast cancer. It is consistent with the previous other studies about its role in most cancers, which indicated that CDKN2B-AS1 exerted a pro-cancer effect [40–42]. For examples, in renal clear cell carcinoma, lncRNA CDKN2B-AS1 affected the malignancy through epigenetically activating NUF2 (NUF2 Component of NDC80 Kinetochore Complex) transcription [16]. Besides, CDKN2B-AS1 had a stimulative function in osteosarcoma and facilitates lung cancer development through their respective ways of regulation [43]. From the views of most reports, their conclusions about the role of lncRNAs CDKN2B-AS1 agreed with our findings and further verified that our results are reliable as well. In summary, in breast cancer, it should be considered lncRNA CDKN2B-AS1 to act as an oncogenic factor.

Similarly, the role of miR-122-5p in breast cancer were further revealed that they took part in the development of breast cancer. Based on our results, depressing the level of miR-122-5p could alleviate the inhibition of knockdown CDKN2B-AS1. In addition, it increased the malignancy of MCF7 and T47D tumor cells with miR-122-5p inhibitor alone. Therefore, we considered that miR-122-5p played a tumor suppressor in breast cancer. However, more evidence is needed to support this view despite there have been articles reported miR-122-5p functions as anti-oncogene in other cancers. Meanwhile, we subsequently verified the STK39 was identified as a target of the miR-122-5p as well. Previous studies have indicated that STK39 accelerates the development of most cancer, such as hepatocellular carcinoma, osteosarcoma and NSCLC [34,35,44,45]. More importantly, several reports have revealed that STK39 promoted progression of breast cancer [39]. For instance, in breast cancer, Li C reported that STK39 regulated by miR-299-5p promoted cell metastasis [39]. These researches uncovered the biological roles of miR-122-5p and STK39 in malignancy are accordance with our results.

Notwithstanding, there are still a few limitations in this work. Apart from the roles of the factors we revealed, more unknown biological functions are still need to be investigated about CDKN2B-AS1 and miR-122-5p. They may play a synergistic role in breast cancer through multi-level regulation. However, this work just uncovered the interaction between the CDKN2B-AS1 and miR-122-5p. Meanwhile, whether CDKN2B-AS1 regualting other molecules such as enzymes or kinases, which participate in tumor progression, is also worth studying.

## 5. Conclusion

In conclusion, these data support our hypothesis that lncRNA CDKN2B-AS1 acts as a miR-122-5p sponge to regulate the STK39 expression, and promotes breast cancer progression.

## Supplementary Material

Supplemental Material

## Abbreviations

BC: breast cancer; lncRNAs: long non-coding RNAs; ceRNA: competing endogenous RNA; CDKN2B-AS1: CDKN2B Antisense RNA 1; miRNAs: MicroRNAs; RISC: RNA-induced silencing complex; HCC: hepatocellular carcinoma; NSCLC: non-small cell lung cancer; STK39: Serine/Threonine Kinase 39; MAPK: mitogen-activated protein kinase; IHC: immunohistochemistry; NUF2: NUF2 Component Of NDC80 Kinetochore Complex; HER2: human epidermal growth factor receptor 2; TN: triple-negative.
