# Supplementary material for: Knockdown of long non-coding RNA CDKN2B-AS1 suppresses the progression of breast cancer by miR-122-5p/STK39 axis
Source: Bioengineered. 2021 Aug 10;12(1):5125–37. doi: 10.1080/21655979.2021.1962685 (PMC8806778; doi:10.1080/21655979.2021.1962685)
Supplement: Supplemental Material [file KBIE_A_1962685_SM9533.zip › Supplementary Material.docx]

**Supplementary Material**

**Knockdown of CDKN2B-AS1 had little impact on the cell cycle of MCF7 and T47D cell.**

As shown in Supplementary Material, we detect the cell cycle of MCF7 and T47D after knocking down of the CDKN2B-AS1. The results indicated that the effect of knockdown of CDKN2B-AS1 on the cell cycle was not statistically significant. Thus, we considered that knockdown of CDKN2B-AS1 had little impact on the cell cycle of MCF7 and T47D cell.

**Knockdown of CDKN2B-AS1 had little impact on the cell cycle of MCF7 and T47D cell.** Flow cytometry analysis of the cell cycle of MCF7 and T47D cells.
